# Pandemic-related financial hardship and disparities in sugar-sweetened beverage consumption and purchasing among San Francisco Bay Area residents during COVID-19

**DOI:** 10.1016/j.pmedr.2022.101759

**Published:** 2022-03-08

**Authors:** Richard Pulvera, Emily Altman, Lizette Avina, Hannah Thompson, Dean Schillinger, Kristine Madsen

**Affiliations:** a School of Public Health, University of California, Berkeley, CA, USA; b Division of General Internal Medicine at San Francisco General Hospital, University of California San Francisco, San Francisco, CA, USA; c Center for Vulnerable Populations at San Francisco General Hospital, University of California San Francisco, San Francisco, CA, USA

**Keywords:** Sugar-sweetened beverages, Health behavior, Diet, Financial stress

## Abstract

Some reports suggest that the COVID-19 pandemic resulted in shifts to unhealthier diets. These unhealthier diets may include sugar-sweetened beverages (SSBs), which strongly contribute to diabetes and other chronic diseases. Using cross-sectional surveys in the San Francisco Bay Area, California, USA we sought to assess self-reported SSB consumption during the pandemic’s shelter-in-place and self-reported changes in SSB purchasing from before to during the pandemic’s shelter-in-place, stratifying by indices of pandemic-related financial hardship. Nearly 60% of our diverse sample (N = 943) reported that it was harder to pay for basics (like food and utilities) during shelter-in-place. Among those who found it harder to pay for basics and received financial assistance during shelter-in-place, we found a ten-fold higher frequency of daily SSB consumption compared to those not facing new financial hardship (2.76 [95% CI: 1.78, 3.74] versus 0.30 [95% CI: 0.23, 0.37] times/day). There were no statistically significant increases in reported purchasing of any SSB, but those with new financial hardship during shelter-in-place reported greater purchasing of regular soda relative to those with no new hardship (0.20 on a 3-point scale [95% CI: 0.03, 0.37]). Our findings suggest that new hardship may increase unhealthy behaviors and worsen existing disparities in SSB consumption. Such disparities are a reminder of the urgent need to reduce economic inequity and improve the quality of our emergency food system in order to mitigate the impact of public health crises like the COVID-19 pandemic.

## Highlights

• Financial hardship among a sample of San Francisco Bay Area residents increased since COVID-19.
• People with financial hardship reported consuming more sugar-sweetened beverages.
• No increased purchasing of sugar-sweetened beverages overall since COVID-19.
• People with new financial hardship increased purchasing of regular soda.

## 1 Introduction

Disruptions stemming from the COVID-19 pandemic have dramatically impacted the financial stability of households (Fox and Bartholomae, 2020), with nearly 40% of lower-income households reporting new financial hardship during the COVID-19 pandemic (Cantor and Landry, 2020). These pandemic-related disruptions have also been associated with changes in lifestyle behaviors. Increased stress during the pandemic has been associated with greater eating and alcohol consumption and increased added-sugar intake (Cummings et al., 2021), as well as increases in other unhealthy, addictive behaviors, such as tobacco use and vaping, among U.S. adults during shelter-in-place (Zhang et al., 2021). The impact of pandemic-related stressors on lifestyle and dietary behaviors is especially concerning for low-income adults, roughly 44% of whom reported being food insecure in mid-March 2020 (Wolfson and Leung, 2020). The COVID-19 pandemic could magnify existing disparities based on income, including differences in sugar-sweetened beverage (SSB) consumption; despite long-term declines in recent years, SSB consumption remains high among low-income U.S. adults (Bleich et al., 2018, Zagorsky and Smith, 2020). Surveying residents of the San Francisco Bay Area, our study sought to explore whether new financial hardship was associated with reported SSB consumption or reported changes in SSB purchasing during shelter-in-place.

## 2 Methods

This cross-sectional study recruited adults (age 18 years or older) living in Berkeley, Oakland, Richmond, and San Francisco, California, USA to complete an online survey about their SSB consumption and purchasing patterns and the impact of pandemic-related stressors. We sought to recruit a sample similar to those from prior in-person street intercept surveys about SSB consumption, conducted from 2014 to 2019 in San Francisco Bay Area low-income neighborhoods (Altman et al., 2021, Lee et al., 2019). Because shelter-in-place orders precluded in-person recruitment, we used address-based sampling and Facebook advertisements to recruit participants to complete an online survey between November 2020 and February 2021. We mailed postcards to a random sample of 8,000 households from the most frequently reported zip codes from prior studies and launched advertisements on Facebook in catchment areas for the same zip codes (Lee et al., 2019). Surveys, available in English or Spanish, took approximately 15 min to complete. A $5 electronic gift card was provided as an incentive.

To assess consumption, we adapted the previously validated 15-item Beverage Questionnaire (Hedrick et al., 2012) for online survey administration. Participants were asked, “About how often do you drink…” for each of: regular soda, energy drinks, sports drinks, sweetened coffee/tea, and fruit drinks. As detailed in the Supplement, we conducted a validation survey via video-call with a subsample of 51 participants to compare SSB consumption as reported in the online versus researcher-administered versions of the survey. Changes in purchasing of regular soda, sweetened fruit drinks, and sports drinks were assessed with: “Since the beginning of shelter-in-place, have you changed the amount of [SSB] that you buy?” on a 5-point Likert scale from “Buying a lot less” to “Buying a lot more”, with a separate “Don’t Buy” response option. For analysis, we collapsed the 5-point scale to a 3-point scale and re-centered this scale to have 0 indicating “No change.”

We assessed ability to pay for basics, on a scale from 1 [not at all harder] to 5 [much harder], with the question: “Since the beginning of shelter-in-place, how much harder is it for you to pay for basics like food, housing, medical care or medicine, and utilities?” We also asked about receiving in-kind food assistance: “…have you gotten free groceries from a food pantry, food bank, church, school, work, or other place that helps with free food?”, and financial assistance: “have you received SNAP, WIC, or vouchers specifically for food?”, with response options “Yes” or “No.” Because securing financial assistance may represent a higher level of need than receiving in-kind food assistance, we created separate indices for financial assistance and in-kind food assistance. Each index (financial assistance or in-kind assistance) was composed of three levels: those who did not report that it was harder to pay for basics regardless of receiving that type of assistance; those who reported that it was harder to pay for basics, not receiving that type of assistance; and those reported that it was harder to pay for basics and receiving that type of assistance. All aspects of this study received ethical approval from the Committee for Protection of Human Subjects (Institutional Review Board) of the University of California Berkeley.

We converted all beverage consumption responses to daily frequencies (times per day) and determined total SSB consumption by summing daily frequencies for the 5 SSB categories (Lee et al., 2019). Generalized linear models with a log link function and a gamma distribution (accounting for the non-negative and right-skewed nature of count data) modeled mean frequency of daily SSB consumption by level of financial hardship. Among those who reported purchasing SSBs, we conducted linear regressions to compare self-reported changes in SSB purchasing by level of financial hardship. All models were adjusted with fixed effects for age, race and ethnicity, gender, education, and city of residence. We calculated robust standard errors to correct for heteroskedasticity. We conducted sensitivity analyses for all outcomes excluding participants from Berkeley due to concerns that a large proportion of relatively well-educated participants living in Berkeley may bias our results. All analyses were conducted in Stata/SE 15.1 (StataCorp LLC, College Station, TX, USA).

## 3 Results

The primary analytic sample included 943 participants (Table 1), of whom 555 participants (58.9%) reported that it was harder to pay for basics since the beginning of shelter-in-place. Those reporting new financial hardship were more likely to be younger than 29 (38.2% vs. 21.1%), less likely to be a college graduate (63.8% vs. 81.1%), and more likely to identify as African-American or Black (12.2% vs. 6.8%), Asian (32.8% vs. 26.6%), or Hispanic or Latinx (16.1% vs. 8.6%). Among those reporting new financial hardship, 39.3% reported receiving in-kind food assistance (just over half of whom had received in-kind food assistance prior to shelter-in-place) and 22.9% reported receiving financial assistance (two-thirds of whom had received financial assistance prior to shelter-in-place). 96 participants were classified at the highest level of need for both indices.

**Table 1:** Sample demographics (N = 943).

		**All participants**		**Participants reporting it was not harder to pay for basics during shelter-in-place**		**Participants reporting it was harder to pay for basics during shelter-in-place**	
		**(%, No.)**		**(%, No.)^a^**		**(%, No.)^a^**	
**Characteristic**		**N = 943**		**N = 388**		**N = 555**	
**Age**							

	18–29	31.2%	294	21.1%	82	38.2%	212
	30–39	21.6%	204	23.2%	90	20.5%	114
	40–49	11.3%	107	11.1%	43	11.5%	64
	50–59	13.1%	124	12.9%	50	13.3%	74
	60+	22.7%	214	31.7%	123	16.4%	91
**Gender**							
	Man	32.6%	305	30.8%	119	33.8%	186
	Woman	63.6%	595	67.1%	259	61.1%	336
	Additional gender identities	3.9%	36	2.1%	8	5.1%	28
**Highest level of education**							
	Less than high school	0.5%	5	0.5%	2	0.5%	3
	High school diploma or GED	8.0%	75	4.7%	18	10.4%	57
	Some college	20.5%	192	13.7%	53	25.3%	139
	College graduate or higher	71.0%	665	81.1%	314	63.8%	351
**Race and ethnicity**							
	African-American or Black	10.0%	92	6.8%	26	12.2%	66
	Asian	30.3%	280	26.6%	102	32.8%	178
	Hispanic or Latinx	13.0%	120	8.6%	33	16.1%	87
	White	42.4%	392	53.5%	205	34.5%	187
	Other	4.4%	41	4.4%	17	4.4%	24
**City of residence**							
	Berkeley	37.5%	354	39.6%	154	36.0%	200
	Oakland	25.6%	242	26.7%	104	24.9%	138
	Richmond	13.2%	125	11.3%	44	14.6%	81
	San Francisco	23.6%	223	22.4%	87	24.5%	136
**Began receiving in-kind food assistance^b^**							
	Never	73.4%	693	91.5%	356	60.7%	337
	During shelter-in-place	12.3%	116	3.9%	15	18.2%	101
	Before shelter-in-place	14.3%	135	4.6%	18	21.1%	117
**Began receiving financial assistance^b^**							
	Never	85.3%	805	96.9%	377	77.1%	428
	During shelter-in-place	4.3%	41	0.3%	1	7.2%	40
	Before shelter-in-place	10.4%	98	2.8%	11	15.7%	87

a Hardship in paying for basics during shelter-in-place was assessed by the following question: “Since the beginning of shelter-in-place, how much harder is it for you to pay for basics like food, housing, medical care, and utilities?”.

b To construct the hardship indices for analysis, we considered those who reported it was not harder to pay for basics as a single group. Among those who reported it was harder to pay for basics, we defined two groups depending on their receipt of in-kind food or financial assistance, regardless of timing of receipt of assistance.

In adjusted models, we observed that reported mean daily SSB consumption during shelter-in-place was significantly higher for groups who found it harder to pay for basics compared to those without new financial hardship, in a stepwise fashion (Table 2). Those with no new financial hardship reported consuming SSBs 0.30 times per day [95% CI: 0.23, 0.37] (reference), those who found it harder to pay for basics but did not receive financial assistance reported consuming SSBs 0.70 times per day [95% CI: 0.55, 0.84] and those who found it harder to pay for basics and received financial assistance reported consuming SSBs 2.76 times per day [95% CI: 1.78, 3.74]. We also found that those who found it harder to pay for basics but did not receive in-kind food assistance reported consuming SSBs 0.79 times per day [95% CI: 0.61, 0.98] and those who found it harder to pay for basics and received in-kind food assistance reported consuming SSBs 1.63 times per day [95% CI: 1.15, 2.11]. From our validation survey conducted through video-call (N = 51), we found good reliability for SSB consumption when comparing the online versus researcher-conducted survey (ICC 0.79; see Supplement for details).

**Table 2:** Adjusted mean daily sugar-sweetened beverage (SSB) consumption and adjusted marginal changes in purchasing, by financial hardship status (N = 920).

		**Hardship and in-kind food assistance**					
		Not harder,		Yes harder,		Yes harder,	
		no in-kind food assistance (Ref)		no in-kind food assistance		yes in-kind food assistance	
		*Mean (95% CI)*		*Mean (95% CI)*		*Mean (95% CI)*	
**Mean daily SSB consumption (times/day) (N = 920)**^**a**^		0.30	(0.23, 0.37)	0.79**	(0.61, 0.98)	1.63**	(1.15, 2.11)
**Change in purchasing**^**b,c**^							
	Soda (N = 595)	−0.08	(-0.15, −0.02)	−0.01	(-0.09, 0.08)	0.05	(-0.06, 0.15)
	Fruit drinks (N = 677)	−0.07	(-0.15, 0.01)	−0.01	(-0.09, 0.08)	−0.02	(-0.14, 0.09)
	Sports drinks (N = 570)	−0.14	(-0.21, −0.08)	−0.08	(-0.16, 0.00)	−0.01	(-0.13, 0.10)
		**Hardship and financial assistance**					
		**Not harder,**		**Yes harder,**		**Yes harder,**	
		**no financial assistance (Ref)**		**no financial assistance**		**yes financial assistance**	
		***Mean (95% CI)***		***Mean (95% CI)***		***Mean (95% CI)***	

**Mean daily SSB consumption (times/day) (N = 920)^a^**		0.30	(0.23, 0.37)	0.70**	(0.55, 0.84)	2.76**	(1.78, 3.74)
**Change in purchasing^b,c^**							
	Soda (N = 595)	−0.09	(-0.15, −0.02)	−0.02	(-0.10, 0.06)	0.12*	(-0.03, 0.27)
	Fruit drinks (N = 677)	−0.07	(-0.15, 0.01)	−0.04	(-0.11, 0.04)	0.06	(-0.09, 0.21)
	Sports drinks (N = 570)	−0.14	(-0.21, −0.08)	−0.06	(-0.13, 0.01)	−0.02	(-0.17, 0.14)

** p < 0.01 * p < 0.05 indicates a statistically significant difference from the value for the Reference Group.

a Reported mean daily SSB consumption is presented in times per day (times/day). Results are adjusted with fixed effects for age, race and ethnicity, gender, education, and city of residence.

b Reported change in purchasing was assessed on a 5-point scale (1–5) which was condensed and re-centered to a 3-point scale (-1, 0, 1) where 0 indicates “No change”, a negative value indicates a reported decrease in purchasing, and a positive value indicates a reported increase in purchasing. Results are adjusted with fixed effects for age, race and ethnicity, gender, education, and city of residence.

c Analyses for change in purchasing exclude participants who reported that they “Don't Buy” a particular SSB category, explaining the smaller sample size. Excludes 332 participants who don't purchase regular soda, 248 participants who don't purchase fruit drinks, and 356 participants who don't purchase sports drinks.

In adjusted models, we assessed reported changes in purchasing among participants who reported purchasing SSBs (N = 595 for regular soda, N = 677 for fruit drinks, N = 570 for sports drinks). We detected no statistically significant increases in purchasing of any SSB overall or within groups (Table 2), although there was a trend towards increased purchasing of soda among those with new hardship receiving financial assistance (0.12 [95% CI: −0.03, 0.27]). In contrast, those without new hardship reported decreased purchasing of regular soda and sports drinks (-0.1 on a 3-point scale; Table 2). Relative to those without hardship, the reported change in purchasing of soda among those with hardship receiving financial assistance was statistically significant (relative difference 0.20 [95% CI: 0.03, 0.37). Our sensitivity analyses excluding participants living in Berkeley yielded similar findings (Supplemental Table 3).

## 4 Discussion

Nearly 60% of our sample found it harder to pay for basics during shelter-in-place and we found a ten-fold difference in reported SSB consumption between those with the greatest financial hardship during shelter-in-place and those with no new hardship. Several studies have documented higher SSB consumption among low-income individuals compared to their high-income counterparts (Han and Powell, 2013, Rehm et al., 2008, Welsh et al., 2011). A recent study using a nationally-representative sample found that individuals in the bottom decile of income and wealth drank 2.5 more SSBs per week than adults in the top income and wealth decile (Zagorsky and Smith, 2020). We document a much larger difference of almost 2.5 SSBs per day between the lowest and highest categories of financial hardship in our sample, recruited from a region with a highly diverse socioeconomic composition.

We found that, relative to those without hardship, reported purchasing of regular soda increased among those with new financial hardship; thus, the large difference in SSB consumption we demonstrate could indicate that new financial hardship and pandemic-related stressors amplified existing disparities in SSB consumption. Given the large reliance on in-kind food assistance in our study (reported by nearly 40% of our sample), it is possible that participants were also receiving SSBs through food assistance programs. There has long been concern about the nutritional quality of the emergency food system, which continues to provide both healthy and unhealthy food and beverage options to families in need (Handforth et al., 2013). The high prevalence of increased hardship during shelter-in-place and increased reliance on food programs highlight the critical nature of fixing the emergency food system. Shocks like the pandemic will have larger health impacts than just COVID-related disease if they also lead to unhealthy diets. Further, to reduce diet-related health inequities (in and out of pandemic circumstances), we should eliminate targeted marketing of SSBs to low-income populations and to children, as other countries have already done (Taillie et al., 2020). Additional interventions that address commercial determinants of health, such as front-of-package labeling, may further reduce disparities in SSB consumption (Gupta et al., 2021, Roberto et al., 2021).

External validity of our findings is limited by our study’s reliance on a convenience sample of participants. Because shelter-in-place restrictions necessitated online survey administration, we were unable to reach residents with limited access to a computer or smartphone. As a result, our sample’s characteristics do not mirror the demographic distribution of samples from previous years (Lee et al., 2019), precluding a longitudinal analysis of changes in SSB consumption. We did not collect data on the participant’s role in obtaining household groceries nor on household size, and we only asked about purchasing of soda and sports and fruit drinks. However, our oversampling of zip codes with higher proportions of low-income residents produced a diverse sample with respect to financial burden as assessed by receipt of in-kind food and financial assistance. Bias from self-reported responses may also impact our study findings. Despite these limitations, this study offers important findings on the relationship between financial hardship and SSB consumption and purchasing.

In our study sample, we find a high prevalence of new financial hardship and high levels of SSB consumption among those facing new financial hardship and receiving in-kind or financial assistance during shelter-in-place. Whether these findings represent pre-existing but previously undocumented levels of disparity in SSB consumption or worsening disparities during shelter-in-place, they are cause for alarm. Our findings emphasize the need to prioritize addressing economic inequity in public health research and advocacy to minimize the impact of shocks like the pandemic. Additionally, we must work to improve the nutritional quality of the emergency food system, to ensure that our “safety net” (including food banks and other food assistance programs) mitigate, rather than exacerbate, the long-term health impacts of pandemics.

## Funding

This study was supported by funding from the National Institutes of Health NIDDK: R01 DK116852 and P30 DK092924.

### CRediT authorship contribution statement

**Richard Pulvera:** Conceptualization, Methodology, Formal analysis, Investigation, Writing – original draft. **Emily Altman:** Conceptualization, Validation, Writing – review & editing. **Lizette Avina:** Validation, Investigation, Writing – review & editing. **Hannah Thompson:** Validation, Writing – review & editing. **Dean Schillinger:** Writing – review & editing, Funding acquisition. **Kristine Madsen:** Conceptualization, Methodology, Writing – review & editing, Supervision, Funding acquisition.

## Declaration of Competing Interest

The authors declare that they have no known competing financial interests or personal relationships that could have appeared to influence the work reported in this paper.
