# Supplementary material for: Pandemic-related financial hardship and disparities in sugar-sweetened beverage consumption and purchasing among San Francisco Bay Area residents during COVID-19
Source: Prev Med Rep. 2022 Mar 8;26:101759. doi: 10.1016/j.pmedr.2022.101759 (PMC8917297; doi:10.1016/j.pmedr.2022.101759)
Supplement: Supplementary data 1 [file mmc1.docx]

**Pandemic-Related Financial Hardship and Disparities in Sugar-Sweetened Beverage Consumption and Purchasing Among San Francisco Bay Area Residents During COVID-19**

**Supplemental Materials**

Richard Pulvera^1^, Emily Altman^1^*, Lizette Avina^1^,
Hannah Thompson^1^, Dean Schillinger^2,3^, Kristine Madsen^1^

^1^ School of Public Health, University of California, Berkeley, CA, USA
^2^ Division of General Internal Medicine at San Francisco General Hospital,
University of California San Francisco, San Francisco, CA, USA
^3^ Center for Vulnerable Populations at San Francisco General Hospital,
University of California San Francisco, San Francisco, CA, USA

* Corresponding Author

_____________________________________________________________________________________

**SUPPLEMENTAL TEXT**

**VALIDATION SURVEY**

**Objective:** To compare reported frequency of SSB consumption between online and researcher-administered beverage frequency questionnaires.

**Participants:** All participants in the main/parent study (N=943) were asked, in the original online survey, if they were open to participating in further research. An additional small incentive ($5 eGift Card) was provided to those who completed the follow-up validation survey. Supplemental Table 1 describes the sample demographics for the 51 validation study participants.

**Methods:** Research assistants contacted participants who indicated interest in participating in further research to schedule a follow-up survey via video call. The follow-up surveys were the same used in prior street intercept surveys (Lee et al., 2019). During the video call, researchers shared a photo of a visual aid of beverage volumes that had been used in previous years during in-person survey administration.

**Results:** The mean absolute difference between online and research-conducted surveys was calculated for each individual SSB measured: soda (0.07 times/day), fruit drinks (0.09 times/day), sports drinks (0.04 times/day), sweetened coffee/tea (0.09 times/day), and energy drinks (0.012 times/day), and 0.25 times/day for overall SSBs (Supplemental Table 2). ICCs, which account for random and systematic measurement errors and estimate overall reliability of measurements, revealed “excellent” reliability for sport drinks (ICC = 0.92), “good” reliability for overall SSBs (ICC = 0.79) and fruit drinks (ICC = 0.76), “moderate” reliability for soda (ICC = 0.73), and sweetened coffee/tea (ICC = 0.69), and “low” reliability for energy drinks (ICC = 0.11) (Supplemental Table 2). Bland-Altman plots do not suggest systematic bias in assessing differences. However, most of the observations that fell outside the 95% limits of agreements were high consumers of the individual SSBs, suggesting that data for high consumers may be less valid (Supplemental Figure 1).

**SENSITIVITY ANALYSES EXCLUDING BERKELEY**

Supplemental Table 3 shows the results of sensitivity analyses for all outcomes when excluding participants living in Berkeley. We conducted these sensitivity analyses due to concerns that a large proportion of relatively well-educated respondents living in Berkeley would unduly bias our results. Our results in these sensitivity analyses are similar to those of the main analyses, although our finding of between-group differences among those who reported new hardship and received financial assistance is no longer statistically significant (0.07 [95% CI: -0.11, 0.26]; p-value from reference = 0.09). Nonetheless, this still suggests that there exists minimal bias in our study due to a large proportion of relatively well-educated participants living in Berkeley.

**REFERENCES**

Lee, M.M., Falbe, J., Schillinger, D., Basu, S., McCulloch, C.E., Madsen, K.A., 2019. Sugar-Sweetened Beverage Consumption 3 Years After the Berkeley, California, Sugar-Sweetened Beverage Tax. Am. J. Public Health 109, 637–639. https://doi.org/10.2105/AJPH.2019.304971

**Supplemental Table 1. Sample demographics of validation survey participants (N = 51).**

|  |  | **All participants in validation survey** | |
| --- | --- | --- | --- |
|  |  | **(%, No.)** | |
| **Characteristic** | | **N = 51** | |
| **Age** | |  |  |
|  | 18-29 | 29.4% | 15 |
|  | 30-39 | 23.5% | 12 |
|  | 40-49 | 9.8% | 5 |
|  | 50-59 | 15.7% | 8 |
|  | 60+ | 21.6% | 11 |
| **Gender** | |  |  |
|  | Man | 27.5% | 14 |
|  | Woman | 68.6% | 35 |
|  | Additional gender identities | 3.9% | 2 |
| **Highest level of education** | |  |  |
|  | Less than high school | 0.0% | 0 |
|  | High school diploma or GED | 7.8% | 4 |
|  | Some college | 25.5% | 13 |
|  | College graduate or higher | 66.7% | 34 |
| **Race and ethnicity** | |  |  |
|  | African-American or Black | 15.7% | 8 |
|  | Asian | 33.3% | 17 |
|  | Hispanic or Latinx | 19.6% | 10 |
|  | White | 29.4% | 15 |
|  | Other | 2.0% | 1 |
| **City of residence** | |  |  |
|  | Berkeley | 31.4% | 16 |
|  | Oakland | 33.3% | 17 |
|  | Richmond | 7.8% | 4 |
|  | San Francisco | 27.5% | 14 |

**Supplemental Table 2. Reported mean daily SSB consumption (times/day) by measurement type, absolute differences, and intraclass correlation coefficients among those who report any consumption.**

|  | **Online survey** | | **Researcher-conducted survey** | | **Absolute difference** | |  |
| --- | --- | --- | --- | --- | --- | --- | --- |
| **Times/day (N)** | **Mean (SD)** | **Range** | **Mean (SD)** | **Range** | **Mean (SD)** | **Range** | **ICC** |
| Soda (35) | 0.09 (0.20) | 0, 1 | 0.10 (0.21) | 0, 1 | 0.07 (0.18) | 0, 0.90 | 0.73 |
| Fruit drinks (45) | 0.14 (0.35) | 0, 2 | 0.10 (0.19) | 0, 0.86 | 0.09 (0.23) | 0, 1.43 | 0.76 |
| Sport drinks (27) | 0.09 (0.21) | 0, 1 | 0.06 (0.15) | 0, 0.71 | 0.04 (0.09) | 0, 0.40 | 0.92 |
| Sweetened coffee/tea (39) | 0.14 (0.33) | 0, 2 | 0.09 (0.24) | 0, 0.71 | 0.09 (0.24) | 0, 1.43 | 0.69 |
| Energy drinks (9) | 0.14 (0.33) | 0, 1 | 0.03 (0.05) | 0, 0.17 | 0.12 (0.32) | 0, 0.97 | 0.11 |
| Total SSBs (46) | 0.40 (0.90) | 0, 4.45 | 0.29 (0.49) | 0, 2.74 | 0.25 (0.56) | 0, 3.20 | 0.79 |

ICC, intraclass correlation coefficient

N, sample size including those who report consumption of that beverage

**Supplemental Table 3. Adjusted mean daily sugar-sweetened beverage (SSB) consumption and adjusted marginal changes in purchasing excluding participants living in Berkeley, by financial hardship status (N = 575).**

|  |  | **Hardship and in-kind food assistance** | | | | | |
| --- | --- | --- | --- | --- | --- | --- | --- |
|  |  | Not harder, | | Yes harder, | | Yes harder, | |
|  |  | no in-kind food assistance (Ref) | | no in-kind food assistance | | yes in-kind food assistance | |
|  |  | *Mean (95% CI)* | | *Mean (95% CI)* | | *Mean (95% CI)* | |
| **Mean daily SSB consumption (times/day) (N=575)^a^** | | 0.36 | (0.26, 0.46) | 0.75** | (0.52, 0.99) | 1.56** | (0.93, 2.18) |
| **Change in purchasing^b,c^** | | |  |  |  |  |  |
|  | Soda (N = 380) | -0.11 | (-0.20, -0.01) | 0.04 | (-0.08, 0.15) | -0.05 | (-0.18, 0.09) |
|  | Fruit drinks (N = 422) | -0.08 | (-0.19, 0.03) | -0.04 | (-0.15, 0.08) | -0.12 | (-0.27, 0.02) |
|  | Sport drinks (N = 360) | -0.19 | (-0.28, -0.10) | -0.08 | (-0.19, 0.03) | -0.04 | (-0.18, 0.10) |
|  |  | **Hardship and financial assistance** | | | | | |
|  |  | Not harder, | | Yes harder, | | Yes harder, | |
|  |  | no financial assistance (Ref) | | no financial assistance | | yes financial assistance | |
|  |  | *Mean (95% CI)* | | *Mean (95% CI)* | | *Mean (95% CI)* | |
| **Mean daily SSB consumption (times/day) (N=575)^a^** | | 0.36 | (0.26, 0.46) | 0.66** | (0.48, 0.85) | 2.73** | (1.41, 4.05) |
| **Change in purchasing^b,c^** | | |  |  |  |  |  |
|  | Soda (N = 380) | -0.11 | (-0.20, -0.02) | -0.02 | (-0.12, 0.08) | 0.07 | (-0.11, 0.26) |
|  | Fruit drinks (N = 422) | -0.08 | (-0.19, 0.03) | -0.05 | (-0.15, 0.04) | -0.13 | (-0.32, 0.06) |
|  | Sport drinks (N = 360) | -0.19 | (-0.28, -0.10) | -0.07 | (-0.16, 0.02) | -0.03 | (-0.23, 0.16) |

** p<0.01 * p<0.05 indicates a statistically significant difference from the value for the Reference Group.

a Reported mean daily SSB consumption is presented in times per day (times/day). Results are adjusted with fixed effects for age, race and ethnicity, gender, education, and city of residence.

b Reported change in purchasing was assessed on a 5-point scale (1-5) which was condensed and re-centered to a 3-point scale (-1, 0, 1) where 0 indicates "No change", a negative value indicates a reported decrease in purchasing, and a positive value indicates a reported increase in purchasing. Results are adjusted with fixed effects for age, race and ethnicity, gender, education, and city of residence.

c Analyses for change in purchasing exclude participants who reported that they "Don't Buy" a particular SSB category, explaining the smaller sample size. Excludes 195 participants who don't purchase regular soda, 153 participants who don't purchase fruit drinks, and 215 participants who don't purchase sports drinks.

**Supplemental Figure 1. Bland-Altman plots of soda, sport drinks, fruit drinks, sweetened coffee/tea, and energy drinks (N=51).**

**
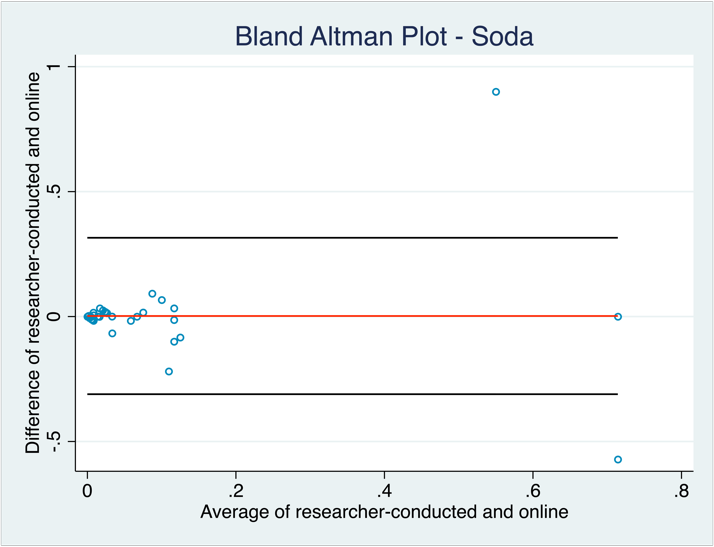

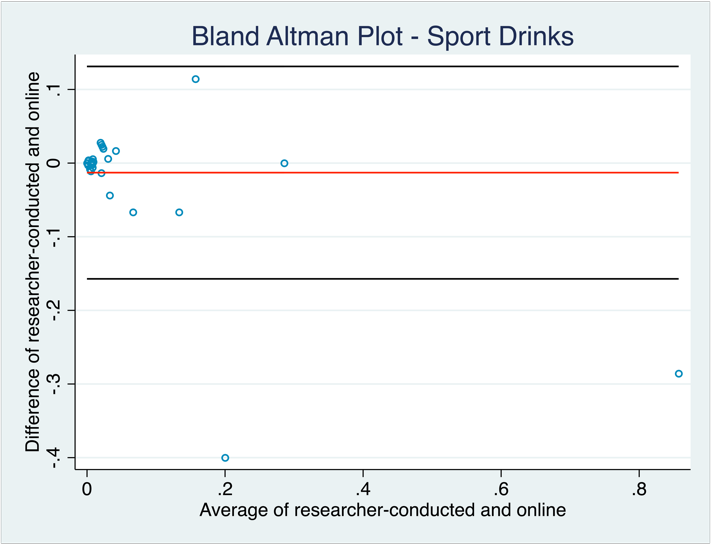
**

**
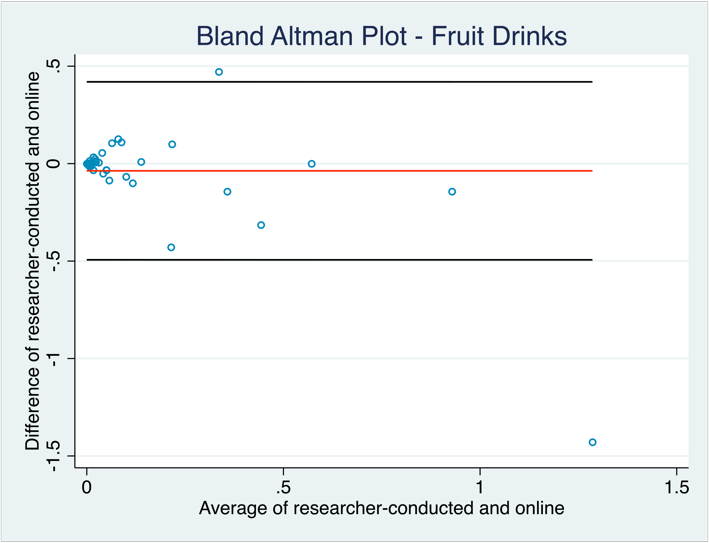

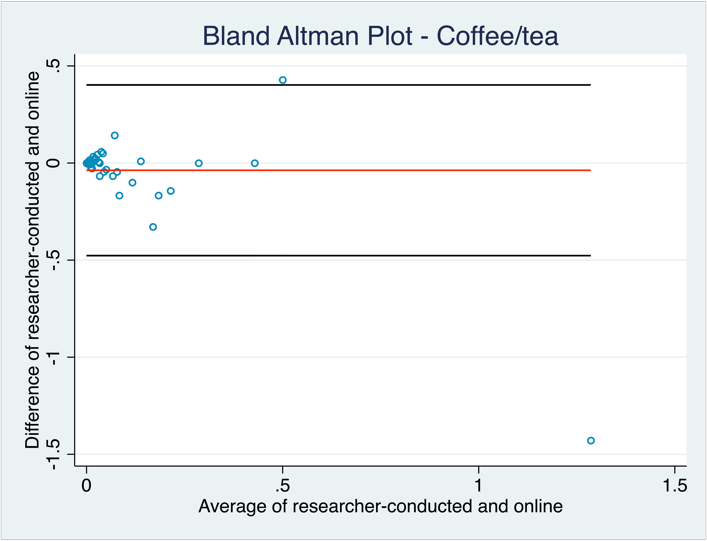
**

**
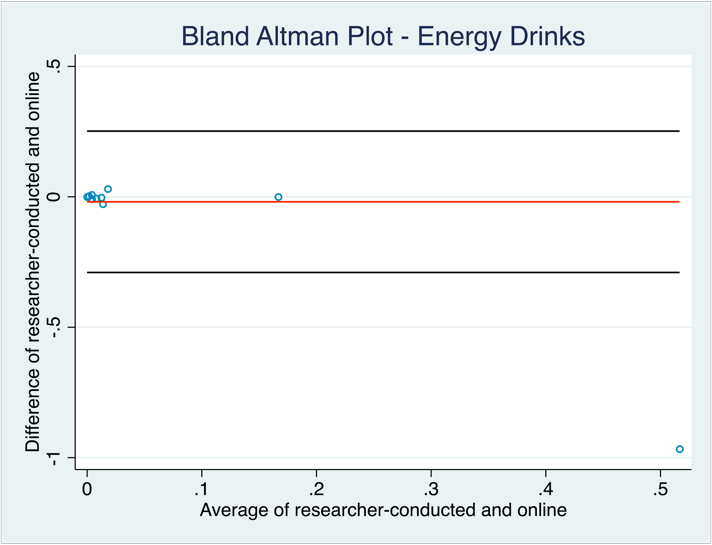

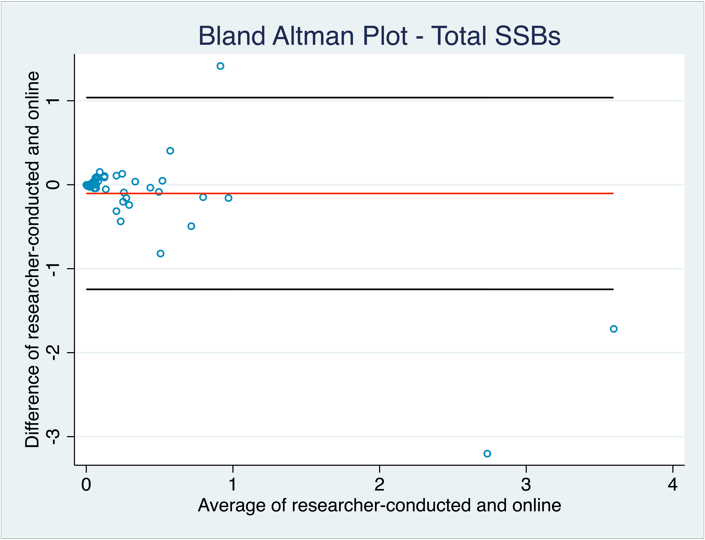
**
